# Muscle differentiation induced up-regulation of calcium-related gene expression in quail myoblasts

**DOI:** 10.5713/ajas.18.0302

**Published:** 2018-05-31

**Authors:** Jeong-Woong Park, Jeong Hyo Lee, Seo Woo Kim, Ji Seon Han, Kyung Soo Kang, Sung-Jo Kim, Tae Sub Park

**Affiliations:** 1 Institute of Green-Bio Science and Technology, Seoul National University, Pyeongchang 25354, Korea; 2 Graduate School of International Agricultural Technology, Seoul National University, Pyeongchang 25354, Korea; 3 Bio Division, Medikinetics, Inc., Pyeongtaek 17792, Korea; 4 Division of Cosmetics and Biotechnology, Hoseo University, Asan 31499, Korea

**Keywords:** Quail, Muscle Differentiation, Calcium, RNA-sequencing

## Abstract

**Objective:**

In the poultry industry, the most important economic traits are meat quality and carcass yield. Thus, many studies were conducted to investigate the regulatory pathways during muscle differentiation. To gain insight of muscle differentiation mechanism during growth period, we identified and validated calcium-related genes which were highly expressed during muscle differentiation through mRNA sequencing analysis.

**Methods:**

We conducted next-generation-sequencing (NGS) analysis of mRNA from undifferentiated QM7 cells and differentiated QM7 cells (day 1 to day 3 of differentiation periods). Subsequently, we obtained calcium related genes related to muscle differentiation process and examined the expression patterns by quantitative reverse-transcription polymerase chain reaction (qRT-PCR).

**Results:**

Through RNA sequencing analysis, we found that the transcription levels of six genes (troponin C1, slow skeletal and cardiac type [*TNNC1*], myosin light chain 1 [*MYL1*], *MYL3*, phospholamban [*PLN*], caveolin 3 [*CAV3*], and calsequestrin 2 [*CASQ2*]) particularly related to calcium regulation were gradually increased according to days of myotube differentiation. Subsequently, we validated the expression patterns of calcium-related genes in quail myoblasts. These results indicated that *TNNC1*, *MYL1*, *MYL3*, *PLN*, *CAV3*, *CASQ2* responded to differentiation and growth performance in quail muscle.

**Conclusion:**

These results indicated that calcium regulation might play a critical role in muscle differentiation. Thus, these findings suggest that further studies would be warranted to investigate the role of calcium ion in muscle differentiation and could provide a useful biomarker for muscle differentiation and growth.

## INTRODUCTION

The most important economic traits in poultry industry are egg production and amount of meat. Thus, genetic selection and breeding programs have made efforts to improve such quantitative traits [1]. Particularly, chicken meat yield is closely related to skeletal muscle growth and so, the understanding of muscle proliferation and differentiation could provide the critical tool for improving the meat amount.

Muscle growth and differentiation in vertebrates including Aves are controlled by a complicated processes. For *in vitro* analysis of muscle growth, the cell culture systems of muscle-originated cells have been established in various species [2]. Many studies on myoblasts which are the precursors of myotubes were conducted to examine the regulatory pathways for muscle differentiation [3]. In Aves, QM7 cell line derived from quail myoblast has been utilized for studying the myogenesis mechanisms [4,5]. In mammals, the onsets of myopathy were caused by the dysregulated myogenic factors such as myogenic regulatory factors (MRFs) [6]. MRFs include a group of four proteins; *MyoD*, *Myf5*, *Myogenin*, and *MRF4* [7,8]. Those MRFs regulate cell cycle arrest of precursor muscle cells and regenerative proliferation. Additionally, MRFs induce muscle differentiation and sarcomere assembly process by activating sarcomere and expressing muscle specific genes [8].

Calcium ion is well known to have various biological functions in body as formation of bone structure, wound healing, and hormone seduction [9]. In muscle, calcium ion acts as a regulatory molecule and signaling molecule in muscle fiber [10]. Generally, calcium signal in muscle is closely related to muscle contraction and muscle relaxation [11]. The muscle contraction pathways regulated by calcium ions show three major mechanisms [11–13]; i) Troponin-tropomyosin complex system which is related to actin-filament in skeletal muscle and cardiac muscle, ii) Myosin light chain kinase mechanism which occurs during muscle contraction with calmodulin in vertebrate’s smooth muscles, iii) Calcium ions bind directly to myosin and induce muscle contraction. Thus, calcium ions are very important for muscle contraction and relaxation but the roles of calcium ions during muscle differentiation have not yet been studied.

Based on mRNA sequencing analysis of quail myoblasts (QM7) during 3 days of myotube differentiation, we analyzed the differentially expressed genes (DEGs) and validated the expression profiles of calcium-related genes to investigate calcium regulatory processes during myotube differentiation.

## MATERIALS AND METHODS

### Quail myoblast culture

According to the previous reports [4,5], QM7 cells (American Type Culture Collection, Manassas, VA, USA) were maintained at 37°C in an atmosphere of 5% CO_2_ and 60% to 70% relative humidity with Medium 199 containing 10% fetal bovine serum (FBS; Invitrogen, Carlsbad, CA, USA), 2% chicken serum (Sigma- Aldrich, St. Louis, MO, USA), and 1× antibiotic-antimycotic (Invitrogen, USA) by subculturing the cells at 70% confluency. To induce myotube differentiation at 90% confluency, the differentiation medium containing 0.5% FBS and 1×antibiotic-antimycotic was changed and half of the medium was replaced daily with fresh differentiation medium.

### Assembly of RNA-sequencing data of quail transcripts

Total mRNAs were isolated from the differentiated QM7 cells during 3 days of myotube differentiation as well as the undifferentiated QM7 cells, and all of samples were triplicated. We generated RNA-sequence data in the QM7 cells. Sequencing of an RNA-sequencing library for each sample was carried out using Illumina HiSeq2500 (Illumina, San Diego, CA, USA) in order to generate 100 pair-end reads. These sequences were aligned and mapped against the chicken reference genome using TopHat for paired-end sequences.

### Total RNA isolation cDNA synthesis

To validate the expression patterns of calcium-related genes, total mRNAs were isolated from the undifferentiated QM7 cells and differentiated QM7 cells (day 1 to day 3 of differentiation periods). The harvested cells were dissolved using 400 μL of TRIzol (Invitrogen, USA) and 100 μL of chloroform was added to remove the organic solvent. After final washing with 75% ethanol, mRNA pellets were dissolved in RNase-free water. The extracted mRNAs were confirmed by measuring the absorbance at 230 nm and 260 nm using a spectrophotometer (NanoDrop 2000, Thermo Scientific, DE, USA) and stored at −70°C for the next experiments.

To synthesize cDNA, 2 μg of RNA, 1 μL of oligo-dT (Invitrogen, USA), and 1 μL of RNase-free water were added and then the mixture was denatured at 80°C for 3 min. Subsequently, the cDNA was synthesized using 4 μL of 5× RT buffer, 5 μL of 2 mM deoxynucleotide (dNTP), 0.5 μL of RNase inhibitor (Promega Corporation, Madison, WI, USA) and 1 μL of Moloney Murine Leukemia Virus reverse transcriptase (Promega, USA).

### Polymerase chain reaction and quantitative polymerase chain reaction amplification

The polymerase chain reaction (PCR) reactions to amplify the target genes in the cDNA were carried out under the following conditions; 1.8 μL dNTP, 2 μL 10× buffer, 0.2 μL Taq, and 12 μL distilled water were added to 2 μL of 50 ng/μL diluted cDNA, and 5 pmol/μL diluted forward primer and reverse primer. The PCR was carried out in a total volume of 20 μL and the PCR procedure was; denaturation at 94°C for 10 min, and a second denaturation at 94°C for 30 s, followed by annealing at 55°C for 30 s and extension at 72°C for 30 s. This step was repeated for 38 cycles and then a final extension was performed at 72°C for 10 min. The band was confirmed on UV light using a 1.5% SeaKem LE agarose gel (Lonza, Rockland, MD, USA). For quantification of calcium-related genes, quantitative reverse transcription-PCR (qRT-PCR) analysis was performed using the iCycler iQ Real-time PCR detection system (Bio-Rad, Hercules, CA, USA) and EvaGreen (Biotium, Fremont, CA, USA). qRT-PCR data of the target genes were normalized relative to beta-actin gene expression and calculated using the 2^−ΔΔCt^ method.

### Bioinformatic analysis

To identify enriched gene ontology (GO) terms, we used DAVID web based bioinformatics tool. A GO enrichment test was performed with a cut-off; raw p value >2.00E, false discovery rate (FDR) >2.5E. In addition, the calcium specific functional cluster was conducted with DAVID. Heat-map visualizations were performed with web-based bioinformatics toll shinyheatmap tool. The amino acid sequences of candidate gene in various species were obtained Ensembl 62. Amino acids were aligned using Multiple Sequence Comparison by Log-Expectation (MUSCLE) (http://www.ebi.ac.uk/ Tools/msa/muscle/).

### Statistical analysis

Statistical analysis was performed using Student t-tests in the SAS software (ver. 9.3; SAS Institute, Cary, NC, USA). Significant differences among the different groups were analyzed using the general linear model in SAS. Differences among treatments were deemed to be significant when p<0.05.

## RESULTS

### Identification of differentially expressed genes during myotube differentiation

**Figure 1:**
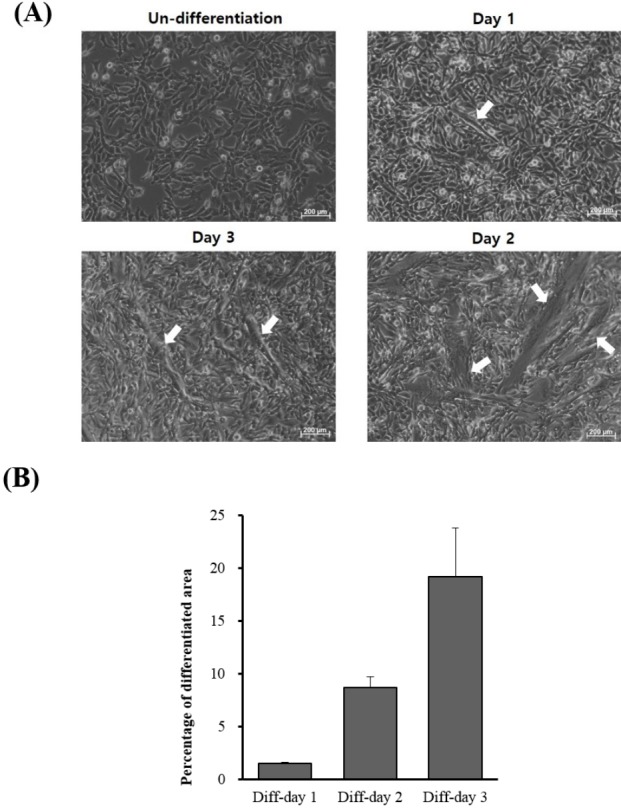
Myotube differentiation of QM7 myoblasts. (A) Morphology of QM7 cells during differentiation periods. QM7 cells transformed into myotubes (arrows) during differentiation. (B) Comparison of differentiated areas during differentiation periods. Percentages of differentiation areas were measured by calculating the occupied myotube area in total areas during myotube differentiation.

**Figure 2:**
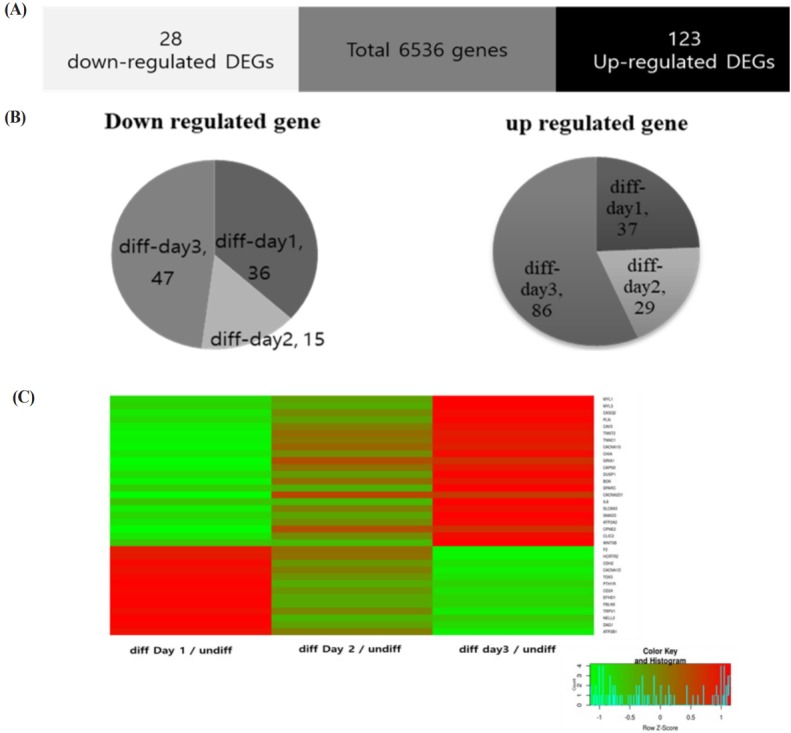
The Venn diagrams and heat map for differentially expressed genes (DEGs) during myotube differentiation. (A) Enhanced DEGs during myotube differentiation in QM7 cells (white bars, >2-fold down-regulated genes; black bars, >2-fold up-regulated genes; gray bars, not differentially expressed). (B) The three pie charts displayed the compositions of the DEGs during myotube differentiation periods (day 1 to day 3) compared to the undifferentiated QM7 cells. (C) Heatmap analysis of calcium-related DEGs during myotube differentiation periods (day 1 to day 3) compared to the undifferentiated QM7 cells. Color from green to red indicates low to high expression.

When QM7 cells were differentiated under the differentiation conditions, QM7 cells were gradually differentiated to myotubes during 3 days (Figure 1A). In addition, the areas of the differentiated myotubes constantly increased according to days of myotube differentiation (Figure 1B). To investigate the muscle differentiation-related genes, we conducted high-throughput RNA sequencing analysis for the undifferentiated QM7 cells and differentiated QM7 cells (day 1 to day 3 of differentiation periods). Based on the sequencing data, we aligned and sorted-out 6,687 genes. Among the expressed transcripts, we calculated the expression levels of all genes from each undifferentiated or differentiated sample (day 1 to day 3 of differentiation periods). In Venn diagram of the comparative analysis, we identified 151 DEGs (cutoff; fold change >2, average of normalized read counts (Log2) >2, p value <0.01) including 123 up- and 28 down-regulated genes (Figure 2A). We also conducted the comparative analysis of DEGs between day 1 to day 3 of differentiation periods and show the Venn diagram in Figure 2B. By comparing with the undifferentiated QM7, 37 and 36 transcripts were identified as up- and down-regulated genes, respectively after day 1 of myotube differentiation. In day 2 of differentiation, 29 and 15 genes were up- and down-regulated. Lastly, 86 and 47 were up- and down-regulated in day 2 of differentiation.

### Functional annotation and selection of calcium-related differentially expressed genes

Based on mRNA sequencing data, we clustered muscle differentiation-related genes and summarized biological process gene ontology of DEGs in QM7 cells after myotube differentiation (Table 1). The most significant enriched terms were ‘skeletal muscle contraction’, ‘negative regulation of calcium ion transmembrane transporter activity’ and ‘branch elongation of an epithelium’ (Fold enrichment = 15.09, p value = 2.17E-03, FDR = 4.93E-02). In the result of biological process GO of DEGs, the calcium ion-related process was one of the significant enriched terms. To investigate calcium ion functions during muscle differentiation, we sorted calcium-related genes based on biological process GO (Table 2). Calcium-specific biological process GO analysis was conducted and 9 calcium-related specific terms were summarized for muscle differentiation genes. As a result, we obtained 35 calcium-related genes in muscle differentiation and compared using Heatmap visualization to examine their expression patterns in each analysis (Figure 2). The expression patterns of calcium-related genes were gradually up- or down-regulated during 3 days of myotube formation (Figure 2).

### Evolutionary analysis and string analysis of calcium-related differentially expressed genes

**Figure 3:**
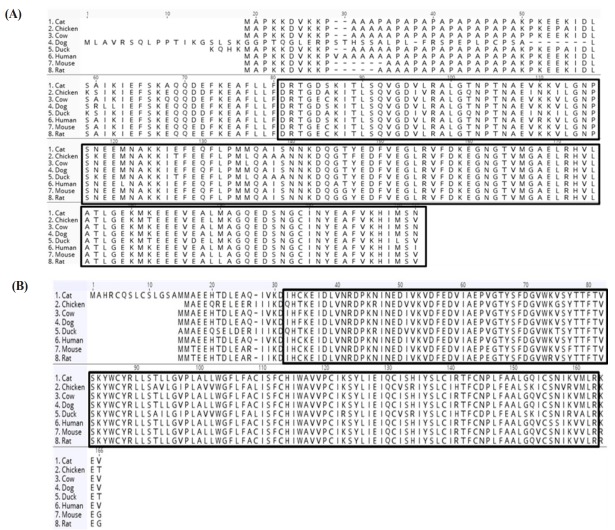
Comparative alignments of amino acid sequences of *MYL1* and *CAV3* gene which were highly expressed in myotube differentiation process. EFh_PEF super family domain of *MYL1* gene (A) and Caveolin domain of *CAV3* gene (B) was highly conserved in various species. The amino acid sequences were aligned by the MUSCLE method in GENEIOUS program. Each conserved domain was marked by solid box. *MYL1*, myosin light chain 1; *CAV3*, caveolin 3.

**Figure 4:**
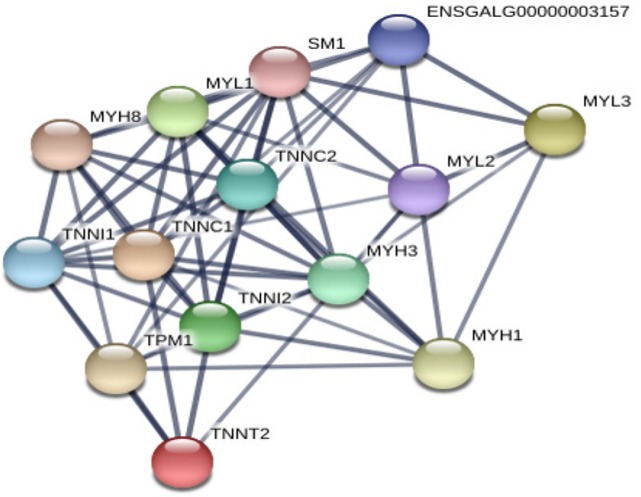
String analysis of calcium-related candidate genes processed by RNA-sequencing data. Protein-protein interactions were analyzed with the STRING software. In the network, proteins were represented as nodes.

To investigate the evolutionary relationships of calcium-related candidate DEGs, we extracted and compared amino acid sequences from eight species in vertebrata (cow, human, duck, mouse, rat, dog, cat, and chicken) regarding two candidate genes, myosin light chain 1 (*MYL1*) and caveolin 3 (*CAV3*). When we conducted multiple alignment with *MYL1* and *CAV3*, EFh_PEF super family domain in *MYL1* and Caveolin domain in *CAV3* showed higher identity (Figure 3, solid box). Therefore, we suggest that calcium-related domains were highly conserved in the candidate genes and these domains would be interactive with calcium ions during muscle differentiation. In addition, we conducted string analysis among seven calcium related candidate genes (Figure 4). As a result, it was confirmed that four genes (*MYL1*, *MYL3*, troponin T2, cardiac type [*TNNT2*], troponin C1, slow skeletal and cardiac type [*TNNC1*]) interacted with each other.

### Validation of calcium-related transcript expression in QM7 cells during myotube differentiation

**Figure 5:**
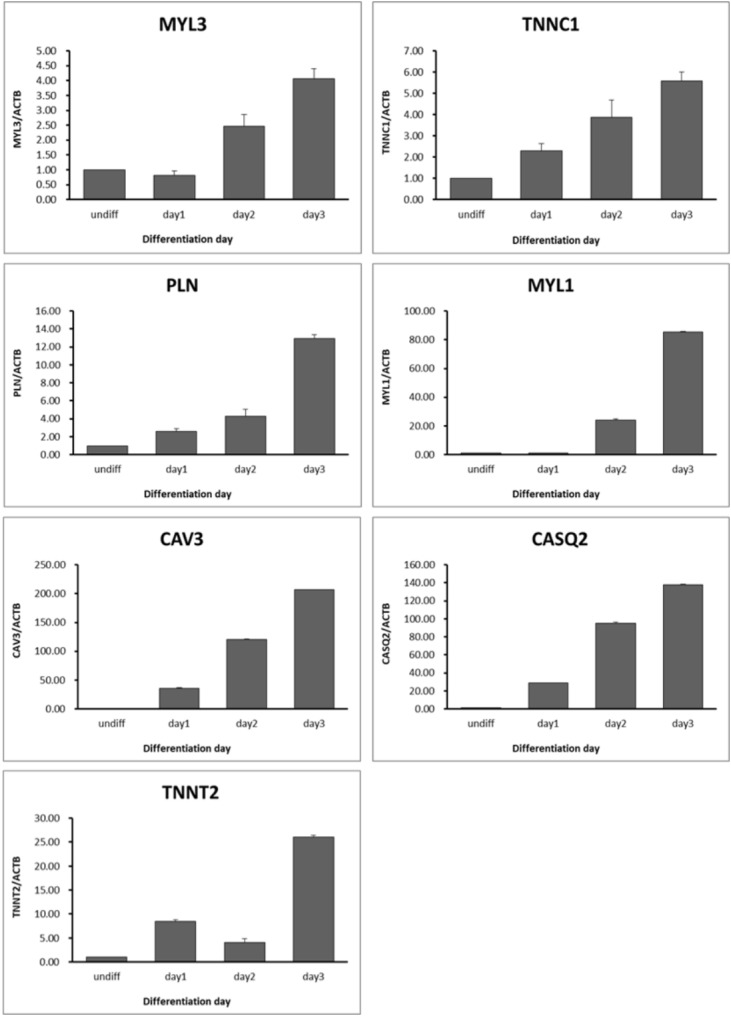
Expression patterns of calcium-related genes during myotube differentiation. Relative expression levels of candidate genes were analyzed by quantitative reverse-transcription polymerase chain reaction (qRT-PCR). (n = 3, p<0.005). Quantitative analysis was performed using the 2^−ΔΔCt^ method by normalization with beta-actin gene.

To validate calcium-related transcript expression in muscle differentiation, we selected and analyzed seven genes which were gradually increased according to days of myotube differentiation periods. Similar to RNA sequencing data, the expression patterns of calcium-related transcripts were gradually and highly increased depending on days of myotube differentiation (Figure 5). The expression level of *MYL3*, *TNNC1*, and phospholamban (*PLN*) transcripts were higher (<20 folds) than those of undifferentiated QM7 cells while the transcription levels of *MYL1*, *CAV3*, calsequestrin 2 (*CASQ2*) were dramatically increased ranging from 90 to 200 fold during muscle differentiation process (Figure 5). These results may indicate that calcium-related genes such as *TNNC1*, *MYL1*, *MYL3*, *PLN*, *CAV3*, *CASQ2*, and *TNNT2* play a critical role in muscle differentiation in QM7 cells.

## DISCUSSION

Cell proliferation, differentiation and cell death are commonly dependent on many signaling pathways controlled by alterations of intracellular calcium [14]. In the previous study, calcium ions were utilized for regulatory molecule and signaling molecule in muscle [10]. It is well-known that calcium plays a role in muscle contraction-relaxation and mechanism of muscle contraction [11]. In addition, many studies reported the biological functions of calcium ions are not only for muscle contraction but also for regulation of energy metabolism through ATP supply [15].

Although the calcium ion plays an important role in muscles, the biological effects of calcium during muscle differentiation have not been well studied. Previous researches indicated that intracellular calcium acted an important role in muscle-specific gene expression, and calcium dependent transcriptional pathway was closely related to skeletal muscle hypertrophic growth [16]. Other studies showed the results of calcium effects on muscle differentiation in frog and rat [17]. In these studies, one of the calcium binding proteins, parvalbumin was identified in myogenesis of frog and rat and detected particularly during the differentiation process of myotomal muscle [18]. Heizmann and Strehler [19] also presented experimental results suggesting the physiological role of parvalbumin in chicken muscle cells. In myotube development of chick embryos, the calcium regulatory system is modulated during myofibiliogenesis and expression level of avian calsequestrin homolog containing calcium storage capacity increased 10-fold before myoblast fusion [20]. These results suggest a biologically significant effect of calcium on muscle differentiation.

In this study, we identified calcium-related genes which presented the up-regulated patterns during myotube differentiation and investigated the binding structure with the highly conserved domains between various species (Figure 3). The expression of *MYL1* gene increased in the myotube differentiation-dependent manner [21] and was significantly detected at an early stage of fast twitch fiber differentiation in zebrafish embryo [22]. The higher similarity of EFh_PEF super family domain sequences in *MYL1* and *CAV3* gene might be evolutionary evidence of muscle formation in vertebrates. When two or more genes have been derived from a common ancestor, these genes are called orthologous genes and contain a similar domain. These orthologous genes may perform a similar function and so could be evolutionary evidence [23]. Calsequestrin (CASQ) is a main calcium binding protein located within the lumen of sarcoplasmic reticulum [24]. A recent report showed that CASQ regulated a RyR channel are closely related to development of skeletal muscle [25]. Particularly, Schwartz and Kay [18] reported that the expression level of CASQ2 considerably increases immediately before myoblast fusion. The regulatory function of TNNC1 (also known as troponin-C type 1) which is one of calcium binding proteins was reported as calcium ion release from the sarcoplasmic reticulum bind troponin subunits (TnC). After binding of calcium ion with troponin subunits, muscle initiates the contraction. Similarly, this modulatory machinery was also observed during myoblast fusion in quail [26]. Collectively, the results presented indicate that calcium-related candidate genes play important roles in avian muscle differentiation.

## Figures and Tables

**Table 1:** Biological process gene ontology (GO) analysis of differentially expressed genes (DEGs) which were specifically up-regulated in myotube differentiation QM7 cells (cutoff; raw p value>2.00E, false discovery rate (FDR) >2.5E)

GO biological process	Expected	Fold enrichment	Raw p value	FDR
Skeletal muscle contraction	0.2	15.09	2.17E-03	4.97E-02
Negative regulation of calcium ion transmembrane transporter activity	0.2	15.09	2.17E-03	4.97E-02
Branch elongation of an epithelium	0.2	15.09	2.17E-03	4.97E-02
Gland morphogenesis	0.85	9.39	7.56E-06	5.80E-04
Positive regulation of endothelial cell migration	0.74	8.13	2.12E-04	8.11E-03
Intrinsic apoptotic signaling pathway in response to DNA damage	0.77	7.83	2.53E-04	9.41E-03
Organ growth	0.91	7.7	8.40E-05	3.86E-03
Cellular response to amino acid stimulus	0.65	7.65	9.15E-04	2.62E-02
Lung alveolus development	0.65	7.65	9.15E-04	2.61E-02
Regulation of morphogenesis of a branching structure	0.65	7.65	9.15E-04	2.60E-02
Cellular response to metal ion	1.02	5.87	9.71E-04	2.75E-02
Myeloid cell homeostasis	1.22	4.91	2.20E-03	4.96E-02
Ossification	2.33	4.72	5.06E-05	2.64E-03
Extracellular matrix organization	2.39	4.61	6.17E-05	3.07E-03
Glial cell differentiation	1.65	4.25	2.05E-03	4.74E-02
Regulation of T-cell activation	2.36	4.24	2.46E-04	9.22E-03

**Table 2:** Biological process gene ontology (GO) terms of calcium-related differentially expressed genes (DEGs) during myotube differentiation in QM7 cells

GO term	Gene count	Gene symbol
Calcium channel complex	10	*CASQ2, CAV3, CACNA1S, CACNA2D1, CACNA1B, CACNA1D, ANXA5, PDE4B, CACNG3, CACNB4*
Calcium channel activity	8	*CAV3, CACNA1S, CACNA1B, CACNA1D, RASA3, TRPV1, SLC24A2, CACNB4*
Calcium mediated signaling	15	*CASQ2, PLN, ATP2A2, CLIC2, HDAC4, CACNA1D, RCAN3, TOX3, SLC9A1, LRRK2, KDR, ATP1B1, TPCN3, TRPV1, GSTO1*
Calcium ion binding	16	*MYL1, MYL3, TNNC1, CHIA, NELL2, DAG1, CAPN1, CDH2, VLDLR, CANX, CIB1, MYL9, ANXA2, EFHD1, SPTAN1, FBLN5*
Calcium ion import	11	*CACNA2D1, ATP2A2, WNT5B, FKBP1A, CACNA1D, RASA3, CHERP, ANXA2, VDAC2, TPCN3, TRPV1*
Calcium ion homeostasis	29	*CASQ2, PLN, CAV3, GRIA1, BOK, SLC8A3, SMAD3, ATP2A2, STIM1, ATP2B1, F2, HCRTR2, CRY2, FKBP1A, RASA3, CHERP, PTH1R, VAPB, CD24, ATP1B1, GNB1, ANXA7, ATP13A2, GALR2, F2RL1, TPCN3, TRPV1, SLC24A2, CACNB4*
Response to calcium	22	*CASQ2, TNNT2, CAPN3, SPARC, CPNE2, STIM1, DUSP1, ENTPD6, ANXA11, ANXA5, IL6, SEC31A, ANXA7, KCNIP2, PPP2CA, CCND1, WNT5A, ALOX5AP, PPIF, RANBP1, SLC25A13, INHBB*
Calcium ion export	2	*SLC8A3, ATP2B1*
Detection of calcium	3	*CASQ2, STIM1, KCNIP2*
